# Functional Monomer Type Determines the Interfacial Properties of Experimental Self-Adhesive Composites Bonded to Dentin

**DOI:** 10.3290/j.jad.c_2378

**Published:** 2025-11-27

**Authors:** Rita Andrade, Carolina Chaves, Diana C. Silva, Ana Paula Serro, Ana Mano Azul, Hugo Águas, António H.S. Delgado

**Affiliations:** a Rita Andrade MSc, Postgraduate Student, Prosthodontics Specialization Program of the Faculdade de Medicina Dentária (FMDUL) of the University of Lisbon, Egas Moniz Center for Interdisciplinary Research (CiiEM), Egas Moniz School of Health and Science, Campus Universitário, 2829-511 Monte de Caparica, Almada, Portugal. Study design, investigation, and drafting the manuscript.; b Carolina Chaves PhD, Assistant Professor of Conservative Dentistry, Egas Moniz Center for Interdisciplinary Research (CiiEM), Egas Moniz School of Health and Science, Campus Universitário, 2829-511 Monte de Caparica, Almada, Portugal. Study design, investigation, and drafting the manuscript.; c Diana C. Silva PhD, Researcher at Instituto Superior Técnico, Universidade de Lisboa; Researcher at Centro de Investigação Interdisciplinar Egas Moniz Center for Interdisciplinary Research (CiiEM), Campus Universitário, 2829-511 Monte de Caparica, Almada, Portugal. Investigation, data curation, and reviewing the manuscript.; d Ana Paula Serro Associate Professor of Biomaterials at Instituto Superior Técnico, Universidade de Lisboa; Researcher at Centro de Investigação Interdisciplinar Egas Moniz, Campus Universitário, 2829-511 Monte de Caparica, Almada, Portugal; Centro de Química Estrutural, Institute of Molecular Sciences and Departamento de Engenharia Química, Instituto Superior Técnico, Universidade de Lisboa, 1049-001 Lisbon, Portugal. Supervision, data curation, and reviewing the manuscript.; e Ana Mano Azul PhD, Associate Professor of Operative Dentistry and Conservative Dentistry at Egas Moniz School of Health and Science; Researcher at Centro de Investigação Interdisciplinar Egas MonizEgas Moniz Center for Interdisciplinary Research (CiiEM), Egas Moniz School of Health and Science, Campus Universitário, 2829-511 Monte de Caparica, Almada, Portugal. Study conception, data curation, and reviewing the manuscript.; f Hugo Águas PhD, Full Professor at Faculdade de Ciências e Tecnologias, Universidade NOVA de Lisboa; CENIMAT|i3N, Department of Materials Science, School of Science and Technology, NOVA University Lisbon, and CEMOP/UNINOVA, Campus de Caparica, 2829-516 Caparica, Portugal. Study methodology, data curation, and reviewing the manuscript.; g António H.S. Delgado PhD, Assistant Professor of Dental Materials and Operative Dentistry at Egas Moniz School of Health and Science; Researcher at Centro de Investigação Interdisciplinar Egas MonizEgas Moniz Center for Interdisciplinary Research (CiiEM), Egas Moniz School of Health and Science, Campus Universitário, 2829-511 Monte de Caparica, Almada, Portugal; Division of Biomaterials and Tissue Engineering, UCL Eastman Dental Institute, London WC1E 6DE, UK. Study design, conception, data curation, statistical analysis, and reviewing the manuscript.

**Keywords:** adhesion to dentin, adhesive materials, Raman spectroscopy, self-adhesive resins

## Abstract

**Purpose:**

To evaluate the influence of functional monomer type and powder-to-liquid ratio on the interfacial properties and degree of conversion of experimental self-adhesive flowable resin composites (SAFRCs) bonded to dentin.

**Materials and Methods:**

Nine experimental SAFRC formulations were developed by varying the powder-to-liquid ratio (1.9, 2.2, 2.5) and the functional monomers included (10-MDP, GPDM, HEMA). Human molars (n = 27) were restored using each formulation and analyzed using a rheometer to assess viscosity, Raman micro-spectroscopy for inter-diffusion zone (IDZ) width, and degree of conversion at the interface (DC%). Two-way ANOVA and post-hoc tests were performed for statistical analysis (α = 0.05).

**Results:**

Rheological testing revealed, as expected, non-Newtonian flow behavior in all composites, with significant effects of both powder-to-liquid ratio (P <0.001) and monomer type (P <0.001) on viscosity. 10-MDP composites exhibited optimal viscosity (1.12–2.86 mPa·s) across all ratios, significantly lower than GPDM and HEMA. Raman mapping showed a distinct IDZ with hybrid characteristics for 10-MDP formulations, contrasting with abrupt transitions or gaps in GPDM and HEMA groups. IDZ width was significantly greater in 10-MDP formulations (P <0.0001). The DC% at the interface was highest for 10-MDP and HEMA formulations, exceeding 68%, while GPDM composites showed lower values (P <0.001).

**Conclusion:**

Functional monomer type critically affects the interfacial bonding performance and conversion rate of SAFRCs, with 10-MDP outperforming GPDM and HEMA in interdiffusion and adhesive quality. Variations in powder-to-liquid ratio influenced viscosity but had a limited impact on interfacial performance. Optimized formulations with 10-MDP may enhance the clinical efficacy of SAFRCs.

**Clinical Relevance Statement:**

Refining acidic-monomer chemistry and viscosity in SAFRCs could improve their bonding predictability.

In response to clinicians’ need for simpler and faster procedures, but also for treatments that seek to enhance patient comfort, manufacturers are investing in new materials aiming to improve the overall effectiveness of dental restorative practice.^4^ It was this point of view that paved the way for the development of self-adhesive flowable resin composites (SAFRCs).^1^ These composites were introduced with the aim of streamlining the restorative procedure, acting by reducing the potential technique sensitivity of the adhesive procedure due to its one-step approach, and logically, its execution time.^1,8^ Their self-adhesiveness relies on the fact that, in addition to their traditional methacrylate monomer systems, they also contain acidic functionalized monomers that follow the adhesion-decalcification mechanism theory initially described by Yoshioka,^53^ allowing them to etch and/or potentially bond to mineralized tooth substrates. Theoretically, these monomers can modify the surface of enamel and dentin, facilitating the creation of microretention zones for subsequent resin impregnation, solubilizing the smear layer, and ideally forming a chemical connection with hydroxyapatite.^8,21,43^ However, current research on available SAFRCs has shown that this is not what actually happens.^44^ These materials have consistently shown poor *in vitro* results and are still far from their ideal goal.^9,18,21,32^

Self-adhesive composites currently on the market are unable to adequately etch dental substrates, dissolve the smear layer, or effectively remove smear plugs from dentinal tubules, resulting in a very limited adhesive potential.^26^ Market options include Fusio Liquid Dentin Flowable Resin (Pentron Clinica, Orange, CA, USA), which contains 4-methacryloxyethyl trimellitate anhydride (4-META) as a functional monomer, Vertise Flow (Kerr, Orange, CA, USA), with glycerol phosphate dimethacrylate (GPDM), and Constic (DMG, Hanau, Germany), which contains 10-methacryloyloxydecyl dihydrogen phosphate (10-MDP).^25,31^ These functional monomers are also present in the materials that are the precursors to SAFRCs – self-adhesive or universal cements.^20^ Almost all commercial SAFRCs also have hydrophilic diluent monomers such as 2-hydroxyethyl methacrylate (HEMA), although unlike 10-MDP or GPDM, this monomer lacks acidic functionality and is added to improve hydrophilicity, wetting, and polymerization kinetics.^5,38^ Compared to conventional adhesive methods, these self-adhesive options exhibit very restricted resin infiltration and minimal demineralization, preventing them from hybridizing dentin effectively.^28^ It has recently been demonstrated that the viscosity of these composites may prevent them from effectively wetting and penetrating the collagen network, an indispensable step to form the hybrid layer.^18^ Furthermore, recent studies have shown that the addition of a functional monomer to these self-adhesive resins does not seem to be enough to guarantee a durable bonding mechanism to the substrate.^15,18^

In summary, tailoring the viscosity of SAFRCs may be a critical factor in enhancing their infiltration capability, particularly in relation to organic substrates such as dentin.^10,11,19^ Additionally, modifications to the functional monomer within the same experimental framework are anticipated to play a pivotal role in maximizing the adhesive potential of these experimental materials. Notably, there is limited research on the interfacial properties of these materials when bonded to dentin, underscoring the need for further investigation in this area.^20^

Thus, the aim of this study was, firstly, to synthesize experimental SAFRCs, with two significant variations: (1) the powder-liquid ratio, which controls the viscosity of the resulting paste, and (2) the type of functional monomer included; and then to qualitatively analyze the chemical composition, to estimate the interdiffusion zone (IDZ) width, and to quantify the conversion rate of these experimental SAFRCs at the composite–dentin interface, using Raman microspectroscopy and *ex-vivo* tooth models. This approach would allow us to determine the impact of the viscosity and/or type of monomer on the bonding profile to dentin of SAFRCs. This investigation tested two null hypotheses: (H_0.1_) varying the powder-to-liquid ratio, and therefore the viscosity, of experimental SAFRCs does not influence their interfacial performance on dentine, as measured by viscosity, IDZ width, and *in-situ *degree of conversion; and (H_0.2_) substituting the functional monomer – 10-MDP, GPDM, or HEMA – has no measurable effect on those same interfacial outcomes.

## MATERIALS AND METHODS

### Samples and Materials

The study sample included 27 sound permanent human molars that were donated (Ethics Committee Process no. 1164). Each tooth was debrided and stored in 1% (v/v) chloramine-T solution for 7 days and subsequently refrigerated at 4ºC.

For this study, nine distinct experimental resin composites were manufactured, with variations in their powder-to-liquid ratio, as well as in the adhesive functional monomer included. The monomers used in this study were 10-MDP from DM Healthcare, San Diego, CA, USA; 2-hydroxyethylmethacrylate (HEMA), obtained from Tokyo Chemical Industry, Tokyo, Japan; glycerol phosphate dimethacrylate (GPDM; DM Healthcare, San Diego, CA, USA), polypropylene glycol dimethacrylate (PPGDMA) from Polysciences (Warrington, PA, USA), and urethane dimethacrylate (UDMA) (Sigma-Aldrich, Schnelldorf, Germany). Camphorquinone (CQ) was obtained from PCM Products (Krefeld, Germany), silica nanoparticles were obtained from Evonik Operations (Essen, Germany), and barium glass filler particles from Dentsply Sirona (Konstanz, Germany).

### Development of the Experimental Composite Formulations

All SAFRC formulations were compounded at the Functional Biomaterials & Polymers Laboratory of Egas Moniz School of Health & Science (Almada, Portugal). Monomers, glass fillers, and initiators described above were obtained from commercial suppliers; none were custom-synthesized. First, the monomer phase was mixed at room temperature in opaque jars. In this mixture, UDMA was weighed and added in the quantities described in Table 1, followed by PPGDMA, which constituted the base mixture. In addition, and separately, 15 mol% of the functional monomer (HEMA, 10-MDP, or GPDM), depending on the experimental group in question, was added to the base mixture. Finally, camphorquinone at 1 wt% was used as the photoinitiator system. Monomers were mixed using a thermal plate at room temperature at 300 rpm for 48 h. The powder phase was prepared using medium-sized barium glass 7 µm (at 90 wt%), and silica nanoparticles (at 10 wt%). The liquid phase was added to the powder in a powder-to-liquid ratio of 2.5, 2.2, or 1.9. Composites were mixed at 1250 rpm for 45 s (DAC 515-200 SE, Flackteck SpeedMixer, Louisville, CO, USA), underwent a preliminary polymerization test, and were stored in an opaque jar at 4ºC.

**Table 1:** Detailed chemical composition of the formulations of experimental SAFRCs used in the present investigation

Group	Composition
**MDP_1.9** **MDP_ 2.2** **MDP_2.5**	**Organic matrix:** 75 wt% UDMA + 24 wt% PPGDMA + 1% CQ (base) + 15 mol% 10-MDP **Inorganic phase:** barium glass (±7 µm) and silica nanoparticles
**GPDM_1.9** **GPDM_2.2** **GPDM_2.5**	**Organic matrix:** 75 wt% UDMA + 24 wt% PPGDMA + 1% CQ (base) + 15 mol% GPDM **Inorganic phase:** barium glass (±7 µm) and silica nanoparticles
**HEMA_1.9** **HEMA_2.2** **HEMA_2.5**	**Organic matrix:** 75 wt% UDMA + 24 wt% PPGDMA + 1% CQ (base) + 15 mol% HEMA **Inorganic phase:** barium glass (±7 µm) and silica nanoparticles


To produce the experimental composites, a molar-based approach was used for each acidic functional monomer included, instead of a fixed mass proportion. This was done to account for variations in molecular weights, as using weight could lead to inconsistent and variable molecule counts, which would not reflect a proper direct comparison between each monomer.

### Rheological Properties

The viscosity of the formulations in the unpolymerized state was tested using a fluidity test on an MCR 92 rheometer, RhemCompass software (Anton Paar, VA, USA), with a cone-plate geometry (CP50), at room temperature. The viscosity curves were obtained at a shear rate of 0 to 100 s-1, with a constant frequency of 10 Hz (n = 3).

### Sample Preparation and Micro-Raman Analyses

Teeth were randomly assigned to nine different experimental groups defined above (Table 1), using a digital algorithm, according to the self-adhesive composite used in the restorative procedure. Each human tooth was sectioned horizontally at the level of the occlusal third, below the cusps, in order to expose the middle coronal dentin, using a hard tissue microtome (Accutom-50, Struers, Denmark), operating at a velocity of 0.350 mm/min. After sectioning each tooth, the smear layer was artificially simulated by polishing the surfaces with 600-grit SiC paper (Buehler, Lake Bluff, IL, USA; LabolPol-4, Struers A/S, Ballerup, Denmark). Teeth were restored with each respective allocated resin composite by applying a 2 ± 0.5 mm layer of composite twice and light-curing each layer for 20 s with an LED light-curing device (950 mW/cm^2^; 450 to 470 nm operating wavelength at zero distance). The average irradiance was monitored every three uses using the Bluelight CheckUp Radiometer device (Bluelight Analytics, USA). After restoration, teeth were stored in artificial saliva^7^ for 24 h in an incubator at 37ºC. Subsequently, teeth were sectioned longitudinally to obtain slices that expose the adhesive interface.

Tooth slices (n = 9) were analyzed by micro-Raman spectroscopy in a Renishaw inViaTM QontorTM confocal Raman Microscope using a 50× magnification objective and a 633 nm laser, focusing on the adhesive interface surface. A Raman mapping analysis was conducted on a surface area of 20 × 20 µm, encompassing the interface, using the 800–1750 cm^-1^ spectral region at a resolution of 2 cm^-1^. The acquisition time was set to 0.5 s, with an accumulation of 5 spectra and the Wire 5.2 software (Renishaw, Wotton-under-Edge, UK) was used to analyze and process the spectra obtained. Post-processing tools included baseline adjustment and subtraction, normalization by normal variation, and noise reduction/smoothening of spectra.

The interdiffusion zone (IDZ) was defined as the resin-infiltrated dentin region extending from the hybrid layer to the point where the mineral and polymer spectra no longer coexist or overlap (as described subsequently using Raman criteria). The IDZ in dentin was estimated by analyzing a random straight line (n = 3), crossing the adhesive interface, and analyzing the variation in intensity of specific peaks, we used the 960 cm^-1^ peak to represent hydroxyapatite (PO₄^3–^) from dentin and the 1457 cm^–1^ peak to represent the CH₂ group of the UDMA monomer from the polymeric matrix of the composite. For this, a Boltzmann function-based approach (Equation 1) was applied to estimate peak intensity data changes over the adhesive interface, following the method outlined by Oliveira Ferraz et al.^30^

Concurrently, the conversion rate (DC %) of monomers during co-polymerization processes was determined, *in situ*, at the interdiffusion zone, by calculating the ratio of the intensity of a reference peak (1457 cm^–1^ for UDMA-based resins) to the reactionary peak (C=C at 1640 cm^–1^) (Equation 2). This was done for both unpolymerized and polymerized samples, based on the method described by Sakano et al.^35^

### Statistical Analyses

All statistical analyses, including descriptive and inferential statistics for testing the null hypotheses, were performed using OriginPro 2023 (OriginLab Corporation, Northampton, MA, USA), with a significance level set at 5%. Peak fittings were also conducted using the same software. A two-way ANOVA was conducted to compare the means obtained in each experimental group. For multiple comparisons in the interdiffusion zone width estimation, Dunn’s post-hoc test was used, whereas the Bonferroni post-hoc test was applied to the conversion rate data comparisons.

## RESULTS

### Rheology

The self-adhesive composites showed contrasts due to the variation in the functional monomer that was used and also due to the powder-to-liquid ratios tested. The viscosity values at a shear rate of 1 s^-1^ are shown in Table 2. Two-way ANOVA revealed that the monomer type variable had a highly significant impact, and with a greater effect size, on the viscosity averages of the composites (two-way ANOVA, P <0.001, η_p_^2^ = 0.93), although the powder-liquid-ratio was also significant (two-way ANOVA, P <0.001, η_p_^2^ = 0.86).

**Table 2:** Viscosity values of non-polymerised experimental composites at a shear rate of 1 s-1. Error bars are ± the standard deviation (n = 3)

Formulation	× 10^5^ mPa·s (1 s^-1^)
MDP_2.5	2.9 ± 0.8
MDP_2.2	1.3 ± 0.7
MDP_1.9	1.1 ± 0.1
GPD_2.5	9.1 ± 0.4
GPD_2.2	4.8 ± 0.4
GPD_1.9	1.6 ± 0.9
HEM_2.5	8.4 ± 0.1
HEM_2.2	4.3 ± 0.8
HEM_1.9	1.4 ± 0.1


Based on the results obtained in the Bonferroni tests (P <0.05), when the monomer factor was considered, HEMA showed no statistically significant difference from the GPDM monomer, while the 10-MDP monomer showed a statistically significant difference from the other two monomers. When the ratio factor (powder/liquid ratio) was considered, there was a statistically significant difference for all the ratios studied.

### Interface Micro-Raman Mapping

**Fig 1:**
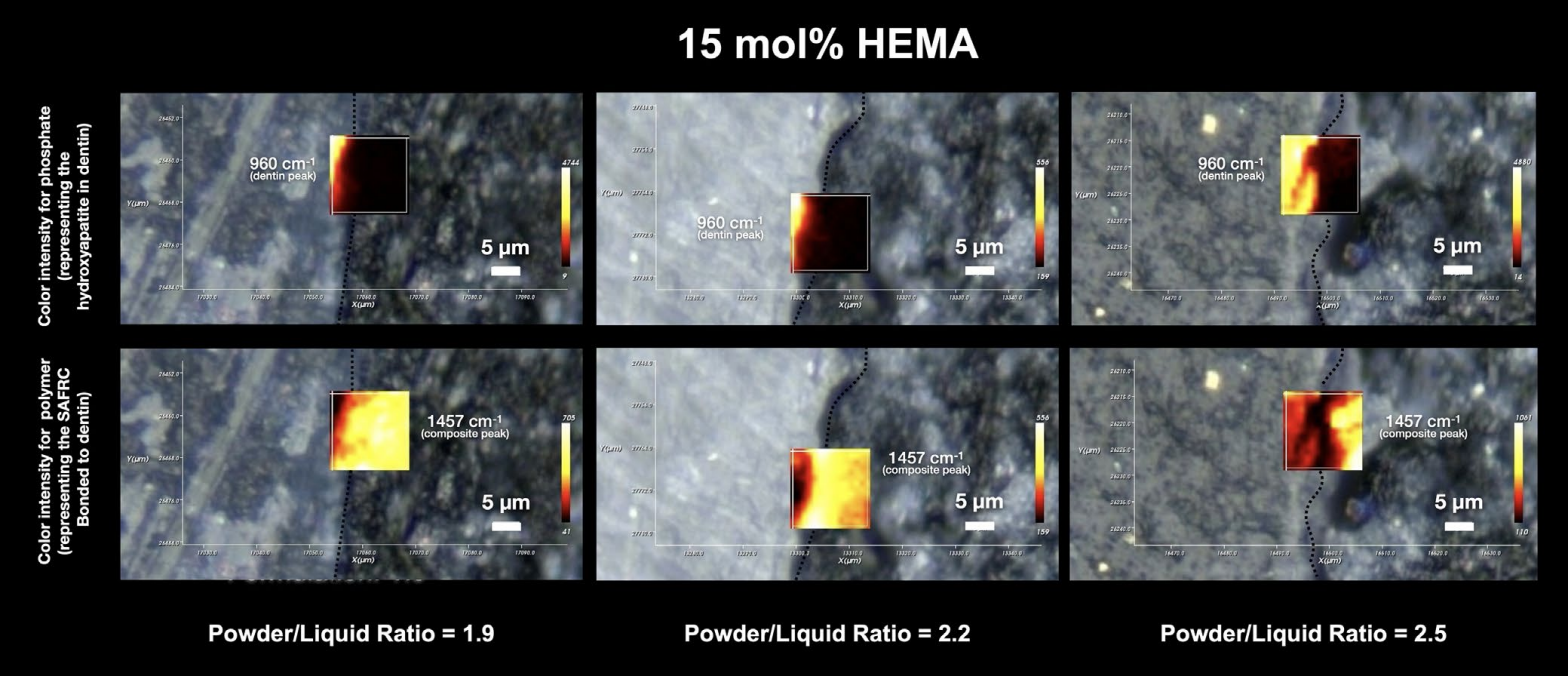
Depiction of intensity maps at the IDZ for different powder-liquid ratios in the HEMA formulation. The dentin contribution (given by the phosphate intensity peak at 960 cm^–1^) and the polymeric material contribution (SAFRCs polymer peak at 1457 cm^–1^) are clearly in two distinct zones, with no observable IDZ where both peak intensities co-exist. At a powder-to-liquid ratio of 2.5, a distinct gap is present, where neither the hydroxyapatite peak nor the polymer peak is detected at the supposed IDZ zone, suggesting a structural flaw.

**Fig 2:**
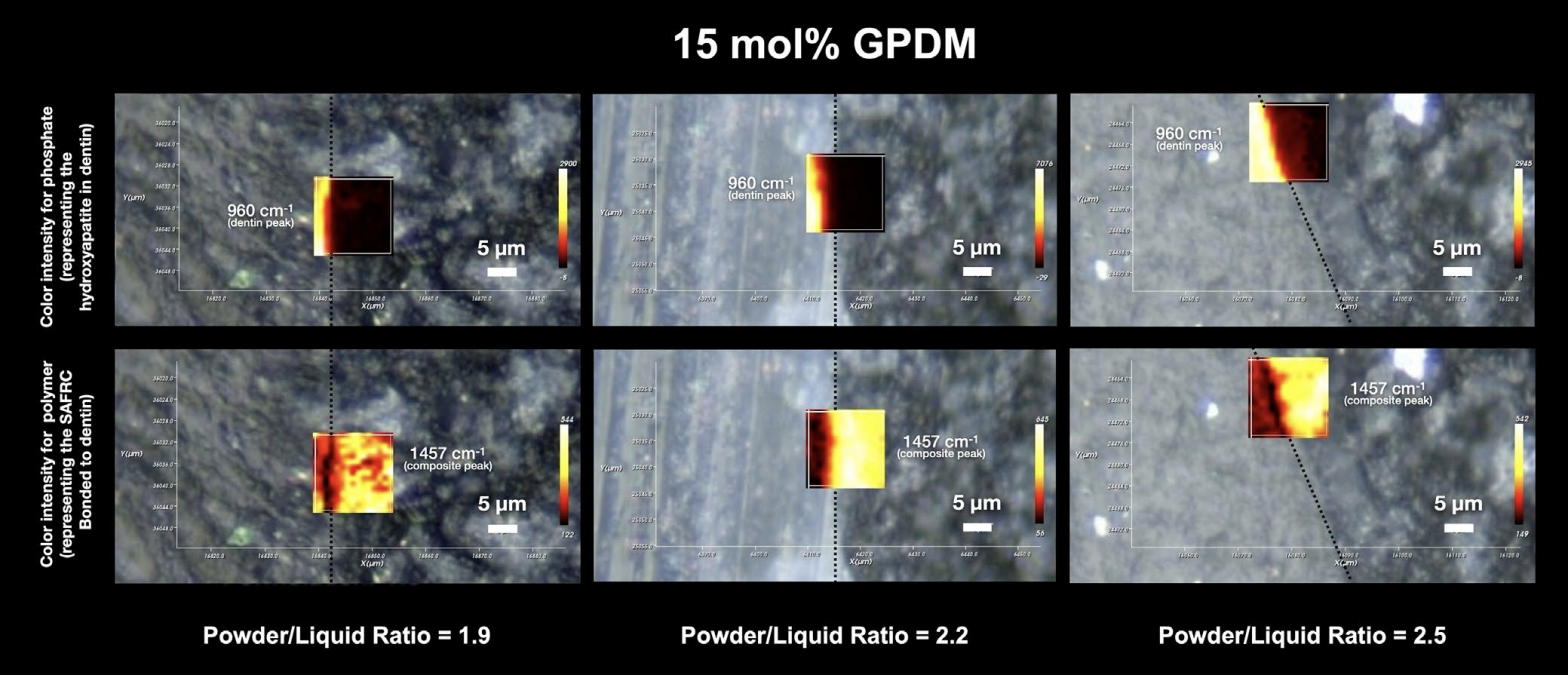
Depiction of intensity maps at the IDZ for different powder-liquid ratios in the GPDM formulation. The dentin contribution (given by the phosphate intensity peak at 960 cm^–1^) and the polymeric material contribution (SAFRCs polymer peak at 1457 cm^–1^) is displayed in this representative image of the overlap at the resin-dentin interface. The intensity of the 1457 cm^–1^ peak and its distribution across the IDZ, together with the phosphate peak, suggest a more prominent IDZ in the 1.9 and 2.5 ratios.

**Fig 3:**
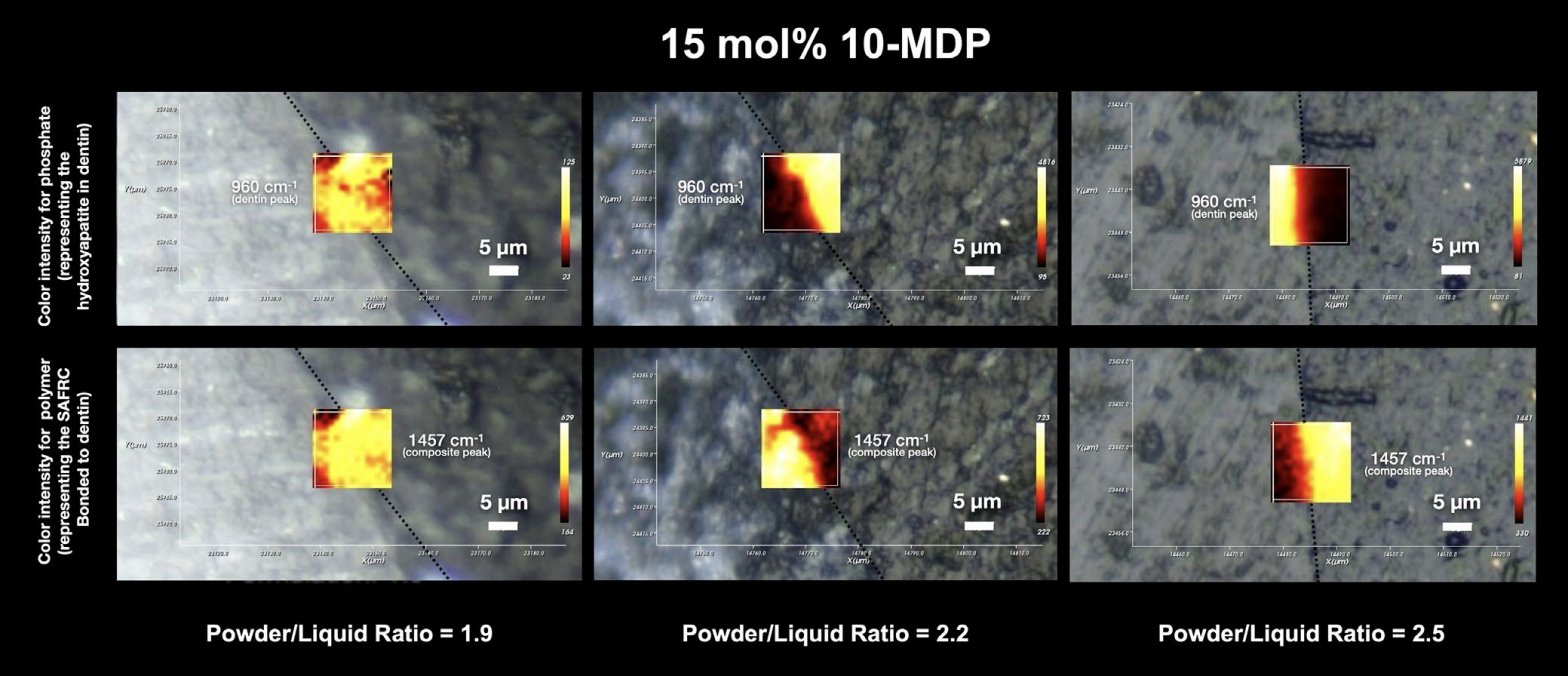
Depiction of intensity maps at the IDZ for different powder-liquid ratios in the 10-MDP formulation. The dentin contribution (given by the phosphate intensity peak at 960 cm^–1^) and the polymeric material contribution (SAFRCs polymer peak at 1457 cm^–1^) is again shown, but with a different chemical profile. It is clear to observe an IDZ in all powder-liquid ratios, with phosphate/polymer peaks co-existing throughout all adhesive interfaces, with strong chemical contributions.

The mapping carried out by Raman micro-spectroscopy on a surface area of 20 µm × 20 µm of the adhesive interface of all the formulations tested can be seen in Figures 1, 2 and 3.

In the maps of the HEMA formulation, the individual contribution of the dentin and the polymeric material can be clearly distinguished, with no clear interdiffusion zone, representing a lack of chemical evidence of the mixture of the two components, with evidence of an abrupt transition or marginal gap. Considering a powder-to-liquid ratio of 2.5, the presence of a gap is visible, where there is no contribution from the hydroxyapatite peak (960 cm^-1^), nor from the peak corresponding to the polymeric material. For GPDM, it is also possible to see a clear distinction between the individual contributions, culminating in the existence of an empty zone, also in the 2.5 formulation (higher viscosity). In the GPDM_1.9 resin formulation, and also in the 2.5 formulation, the presence of an interdiffusion zone seems to be partially more evident, since there is intensity in the contribution of the 1457 cm^-1^ peak at the interface, both in the demineralized dentin zone and in the mineralized part, although it is possible to observe a dark zone, without intensity, at the interface (Fig 3).

**Fig 4:**
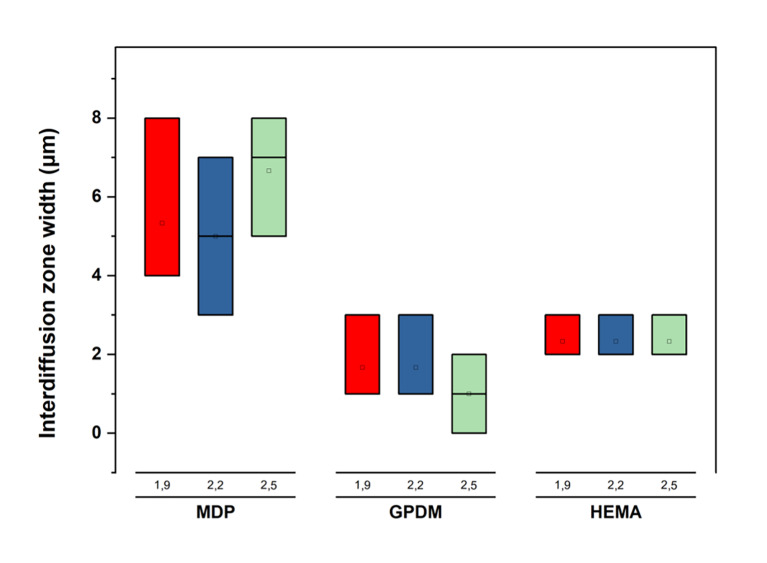
Interdiffusion zone width ranges, illustrating a significantly greater width in the 10-MDP formulations, in all the proportions tested, compared to GPDM and HEMA, as confirmed (Dunn’s test, P <0,05).

Compared to previous functional monomers, the chemical profile of the interdiffusion of 10-MDP formulations is significantly different. In all formulations, there are no empty or discontinuous areas, and there is strong evidence of interdiffusion zones, with a clear representation of the mixture of the two components, which suggests the existence of a hybrid layer. The intensity of the material contribution varies with depth at the interface, but there is a smooth transition of contributions between the dentin and the material. In this material, viscosity does not seem to have induced differences in chemical mapping (Fig 4).

### Interdiffusion Zone Width

Considering the study of the width of the interdiffusion zone, where there is chemical evidence of components of the dentin and the experimental composites at the same time, there were again differences only for the type of functional monomer used (Kruskal–Wallis, x^2^ = 18.8; P <0.0001), since the 10-MDP formulations showed a significantly larger interdiffusion zone. These results are presented in interval form to reduce the error associated with a fixed distance, associated with the resolution of Raman micro-spectroscopy. The intervals can be seen in boxplot form in Figure 4. It is possible to observe that the differences lie in the comparison between MDP and GPDM (Dunn’s test, P <0.0001) and MDP vs HEMA (Dunn’s test, P = 0.01). There were no differences between the experimental composite formulations containing HEMA and those containing GPDM (P = 0.5). Additionally, varying the ratio of powder to liquid was not shown to induce differences in the interdiffusion zone ranges (Kruskal–Wallis, P = 0.99).

### Degree of Conversion at the Interface

**Fig 5:**
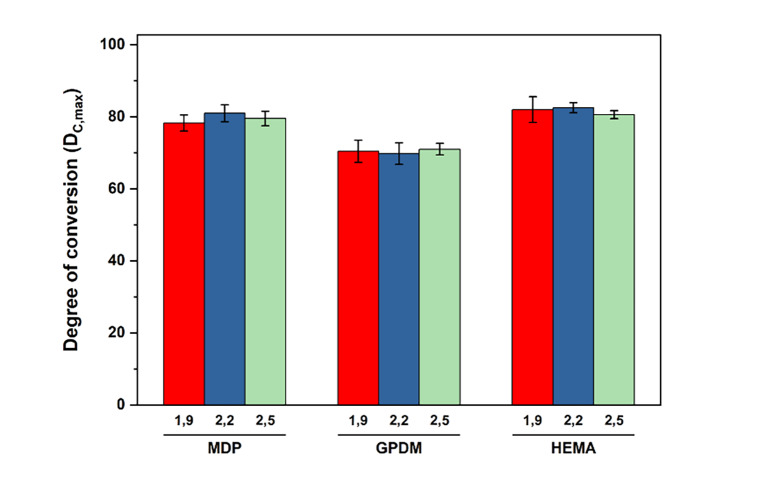

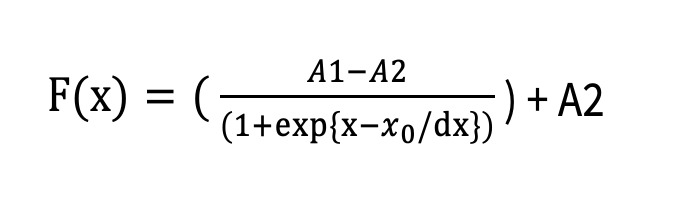

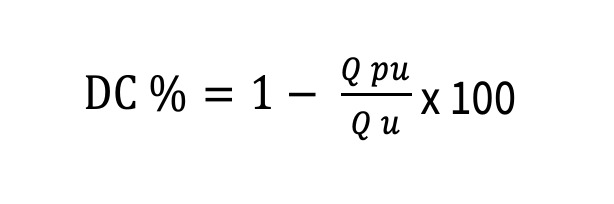
Conversion rate results in a bar graph, with error bars illustrating the standard error of the mean. Differences were found only between formulations containing GPDM versus others, with significantly lower means significantly lower means in the former (GPDM formulations) (P <0.01, Bonferroni).

With regard to the conversion rate (DC %), measured at the adhesive interface, the results of the factorial model applied confirm the impact of the “monomer” variable on the rate (two-way ANOVA, F = 18.6; P <0.0001). A bar chart is shown in Figure 5. In all the formulations, the 10-MDP monomer and HEMA were similar, and there were no significant differences between them. However, compared to GPDM, both formulations containing 10-MDP and HEMA obtained significantly higher conversion rates than GPDM formulations, confirmed by Bonferroni post-hoc (HEMA vs GPDM P <0.0001, and 10-MDP vs GPDM P <0.001).

The variation between powder and liquid ratio did not translate into differences in the conversion rates obtained (P = 0.89), revealing that only the monomer choice had an impact on conversion.

## DISCUSSION

In an effort to establish both chemical and mechanical adhesion without the need for an extra step, there is an increasing interest in creating materials that combine restorative and adhesive qualities in a single composition.^21^ This method may simplify the restorative procedure, thereby increasing its effectiveness and predictability.^16^ However, as their bond strength is still lower than that of conventional adhesives, together with universal composites, the available current data suggest that the adhesive performance of SAFRCs is largely insufficient for the best possible clinical results.^34,39^ In order to overcome the drawbacks of SAFRCs and improve their efficacy, next-generation materials are therefore required.^34^

Research on SAFRCs is limited. The majority of the studies are on commercial self-adhesive composites and focus on the effects of adding new functional particles, assessing bond strength, or changing the photoinitiator system.^27,47,48^ Recently, Nunes Ferreira et al^15^ have demonstrated how different functional monomers affect the rates of polymerization, cell metabolism, and sealing ability in SAFRCs. Concurrently, a previous study has looked at how viscosity affected resin penetration and bond strength to dentin, noting that low viscosity resins had a hindered penetration within the dense collagen network.^18^ These findings suggested that the high viscosity of SAFRCs influenced the spreading of the pastes on the surface of dentin, likely compromising their wettability, consequently preventing any potential interaction between the resin monomers and the substrate.^15,18^ This eventually leads to a compromise in the ability of the monomers to penetrate and properly hybridize the organic matrix in dentin, and effectively contact with hydroxyapatite, essentially required for the establishment of stable chemical interactions.^18,44^ Thus, this study highlights the importance of examining the viscosity of SAFRCs alongside the type of functional monomer to determine their respective impacts on the interfacial quality of the adhesive layer formed in dentin.

The primary objective of this study was to analyze the rheological properties of experimental self-adhesive flowable composites formulated with various functional monomers (HEMA, GPDM, and 10-MDP). The results focused on the viscosity of these materials, tested at different powder/liquid ratios (1.9, 2.2, and 2.5). The data compel the acceptance of the first null hypothesis and the rejection of the second. First, lowering paste viscosity—achieved by reducing the powder-to-liquid ratio – did not significantly broaden the inter-diffusion zone, its quality, and the *in-situ* DC%, thereby accepting H_0.1_. Second, substituting GPDM or HEMA with 10-MDP produced a chemically richer interface, broader IDZ, and a higher gain in DC%, outcomes that likewise contradict H_0._2. These findings confirm that monomer selection is a decisive lever to improve dentine adhesion of SAFRCs and support the broader conclusion that compositional refinement will be required to move SAFRCs beyond their current niche indications. The study revealed that the self-adhesive flowable resins exhibited non-Newtonian behavior, meaning that as the shear rate increased, the viscosity decreased. A similar observation was made in the study conducted by Beun et al,^2^ in which the researchers investigated the rheological behavior of experimental fluid resin composites and evaluated the influence of each component, both organic and inorganic, on their viscoelastic properties, testing experimental formulation models. According to the authors, the complex viscoelastic properties of resin composites are influenced by the interactions between microfiller and monomer molecules. By modifying the chemical and physical properties of the surface of the particles, it is possible to improve their flow properties and clinical handling performance. The authors also found that the viscosity of the materials increased as the content of filler particles increased.^2,22,23^ The viscosity of composites is affected by factors such as the type and proportion of the resin matrix components, as well as the size and shape of the particles.^24^ The interlacing between filler particles and the interface interactions between filler particles and the resin matrix are particularly important.^22^ The study observed that different ratios of monomers resulted in varying viscosities, with ratios of 2.5 showing the lowest viscosity.

The present study demonstrated that the viscosity of the resin composite formulation is dependent on the functional monomer utilized. As stated by Beun et al,^2^ an organic matrix viscosity between 1 Pa·s and 2 Pa·s is optimal for achieving favorable clinical performance and mechanical properties. Viscosity tests demonstrated that all self-adhesive flowable resin composites formulated with 10-MDP, across all selected ratios, displayed viscosities within the optimal range (1.12 to 2.86 mPa·s) as previously outlined. Furthermore, GPDM at a 1.9 ratio (1.61 mPa·s) and HEMA at a 1.9 ratio (1.40 mPa·s) also demonstrated viscosities that fulfilled the criteria for effective clinical performance, as outlined by Beun et al.^2^

The study revealed that experimental resins formulated with the 10-MDP monomer exhibited markedly lower viscosity compared to GPDM and HEMA, which demonstrated no statistically significant differences between them. The viscosity differences are attributable to the molecular structure of the monomers.^29^ 10-MDP comprises two functional groups, namely a phosphate group for bonding with apatite and a copolymerizable methacrylate group, which are separated by a long intermediate chain. This structure enhances hydrophobicity and stability against degradation, thereby outperforming monomers such as 4-META or Phenyl-P.^14^ As posited by Delgado et al,^12^ the elevated solubility of 10-MDP in UDMA/PPGDMA systems, attributable to its extended chain, could exert an influence on viscosity. Despite 10-MDP being added in 15 mol% to all formulations, its phosphate groups may have prompted molecular interactions, thereby potentially affecting viscosity and diffusion rates over time.^52^

The distribution of chemical components in the adhesive interface was analyzed using micro-Raman spectroscopy, a reliable testing method as pointed out by the Academy of Dental Materials.^12,46^ While there are numerous similar studies on cement formulations and adhesive systems, very few have examined this in SAFRCs.^13^ The IDZ for self-adhesive cements has been described to be different to the one produced with adhesives and conventional cements, since it lacks distinctive micromorphological features.^38^ According to the present findings, there was no chemical indication of a well-infiltrated IDZ in the HEMA and GPDM formulations. Rather, evidence of an abrupt change or even a gap at the interface was observed. This had also been previously pointed out in the literature.^11^ Conversely, a clear IDZ containing a hybrid combination of organic dentin counterparts and the polymeric matrix was clearly seen with the 10-MDP formulations, regardless of the viscosity. This may indicate the existence of a hybrid layer. These findings are consistent with laboratory data over the years showing that 10-MDP has an outstanding adhesive performance, particularly in dental adhesives.^33,49^

It is well-established that 10-MDP forms a highly stable and effective bond with hydroxyapatite crystals.^12,33^ The creation of an insoluble 10-MDP-Ca salt, along with its hydrophobic molecular structure, enhances the quality of the adhesive interface and contributes to increased durability and reliability. In contrast, resin composites containing alternative monomers like GPDM or HEMA fail to achieve effective dentin hybridization. Due to its pronounced hydrophilicity, HEMA can lead to early and significant hydrolysis of the adhesive layer.^33^ Additionally, unlike 10-MDP, these alternative monomers cannot form water-insoluble, resilient calcium salts.^12,33^

Micro-Raman mapping images also revealed differences in the viscosities of the experimental GPDM and HEMA. At a powder-to-liquid ratio of 2.5, the imaging results revealed that the gap was more noticeable, suggesting that the composites were further hindered by the increased viscosity to form an effective interdiffusion zone. This hindered the development of a hybrid layer by compromising their capacity to demineralize and penetrate dentin.^28,44^ The contact angle that formed between the composite and dentin substrate may have been too high to allow proper intimate contact.^14^ However, it is important to note that the interdiffusion zone seemed to be unaffected by the viscosity in the 10-MDP formulations, suggesting that the unique chemical structure of this monomer, and its properties, provide important adhesive and cohesive strength at the interface.^13^

These features include: (1) its superior and more stable affinity for hydroxyapatite compared to other monomers^17^; (2) its capacity to form insoluble salts^52^; (3) 10-MDP’s unique equilibrium between hydrophilic and hydrophobic domains enables effective substrate wetting while ensuring adhesive durability and stability^19^; (4) its excellent co-polymerization capacity.^12,36^ Additionally, other factors may have imparted this outcome.^12,51^ In fact, its capacity to create nanolayers of 10-MDP-Ca salts – a process called nanolayering – may help to stabilize the adhesive interface.^42^ Also this monomer forms a stable collagen-phosphate complex through hydrogen bonding with the collagen in dentin.^19^

Interestingly, previous studies have shown that adhesives formulated with HEMA have a significantly narrower interdiffusion zone compared to adhesives that do not include this monomer.^44^ This difference in the width of the interdiffusion zone is probably influenced by the viscosity of the adhesives, which is affected by the presence of HEMA.^37^ According to the authors’ hypothesis, the overall viscosity of adhesives containing HEMA may make it more difficult for the adhesive to penetrate the substrate, thus reducing the extent of interdiffusion.^3^ It is important to note, however, that comparing adhesives with composite materials involves fundamentally different considerations since the mechanisms governing their interfacial behavior differ significantly.^10^ Nevertheless, this factor may have influenced the behavior of SAFRCs formulated with HEMA.

Regarding GPDM, the poorer results can be partly explained by the low levels of polymerization seen with this monomer. This has been noted not only in this study but also in earlier investigations.^12,15,50^ It appears that this monomer is more prone to phase separation, which results in the creation of a hybrid layer that is more unstable.^13^ That is, the resin components separate into hydrophobic and hydrophilic phases as the resin demineralizes and diffuses into wet dentin.^40^ The hydrophobic resin monomers cannot form an impermeable three-dimensional polymer that can shield the collagen fibers since they are not free to penetrate the demineralized and moist dentin matrix on their own.^40^

The conversion rate at the adhesive interface was measured in addition to the qualitative analysis. As stated by Cadenaro et al,^5^ polymeric restorative materials only acquire their final properties when the polymer is fully formed. Consequently, efficient polymerization in the hybrid layer translates into greater strength and stability of the entire resin-dentin interface.^5,10,41^ In the present study, all of the experimental self-adhesive composites achieved conversion rates greater than 68%. As previously stated, there is currently no set minimum acceptable conversion rate value for direct restorative materials.^37^ However, to prevent the release of cytotoxic, unpolymerized monomers, a minimum conversion rate of 50% must be reached.^10^ Furthermore, the experimentals containing 10-MDP and HEMA monomers showed the highest conversion rates. With 10-MDP, the conversion rates matched those seen with FT-IR in previous studies with comparable formulations.^13^The conversion rate can be positively impacted by characteristics like intermolecular interactions, flexibility, molecular weight, and the number of double bonds of the molecule.^12,45^ Other reports of comparable formulations reached lower values,^15^ mainly due to the fact that in this study, the chemical matrix was optimized, with the addition of PPGDMA in place of triethylene glycol dimethacrylate (TEGDMA), a more flexible and reactive monomer, which improves polymerization kinetics.^33^ However, the high conversion rate trend seen with HEMA is in line with these earlier studies.^18^ During polymerization, which comes before the crosslinking phase, both HEMA and 10-MDP monomers help to form linear chains.^12,15^ The high conversion rates seen in resins containing these monomers could be explained by this early-stage polymerization feature.^10^ Furthermore, the glass transition temperature of monomers affects the rapidity of their conversion; higher conversion levels are typically linked to lower glass transition temperatures.^6^ Indeed, HEMA is one of the monomers that can achieve high conversion levels before vitrification because of its lower glass transition temperature.^6,12^ Considering the formulations containing GPDM, the lower conversion rates can be attributed to its chemical structure, which affects the chemical interactions necessary for polymerization.^15,50^ The GPDM monomer is bulkier and less mobile compared to other monomers, potentially straining the polymerization reaction, while inducing steric hindrance effects.^50^

## CONCLUSION

The interfacial quality of SAFRCs bonded to dentin is greatly affected by the type of functional monomer included in the formulation, while the powder-liquid ratio of the mixture, which controls the viscosity, has a limited effect. Within the different monomers that were tested, 10-MDP outperformed all others, and such was the case for interdiffusion zone width, chemical quality, and conversion rate. A detailed overlook at the chemical composition of current SAFRCs is required to develop next-generation formulations able to support the upcoming needs of modern restorative.

### Ackowledgments

**Ethics Committee:** Process no. 1164, Ethics Committee of Instituto Universitário Egas Moniz.

This work was supported by the CiiEM Investiga 2022 Grant, awarded by the Centro de Investigação Interdisciplinar Egas Moniz [Grant: SELFINO], Egas Moniz School of Health & Science.

## Supplement

### Equations

**Equation 1:** The Boltzmann adjustment equation, where A1 and A2 are the initial and final plateau of the peak intensity of the band, respectively; x is the displacement across the interface, x0 is the midpoint of the inter-diffusion zone, and dx represents the width.

**Equation 2:** Equation used to calculate the degree of conversion – DC (%) *in situ*, where Q is the ratio of the intensity of the methacrylate peaks (reaction/reference peak) in the polymerized (*p*) and non-polymerized (u) samples.
